# The Biomechanical Behavior of Selected Achilles Tendon Revision Constructs: An Exploratory Cadaveric Study

**DOI:** 10.3390/bioengineering13060594

**Published:** 2026-05-22

**Authors:** Horia-Mihnea Fotescu, Dragoș Apostu, Noémi Mosonyi, Daniel Oltean-Dan, Horea Benea, Dan Cosma, Cosmin Cosma, Xavier Martín Oliva

**Affiliations:** 1 Department of Orthopaedics and Traumatology, University of Medicine and Pharmacy, 400012 Cluj-Napoca, Romaniamosonyi_noemi@yahoo.com (N.M.); olteandandaniel@yahoo.com (D.O.-D.); beneahorea@yahoo.com (H.B.); dicosma@gmail.com (D.C.); 2 Department of Manufacturing Engineering, Technical University of Cluj-Napoca, 400641 Cluj-Napoca, Romania; cosmin.cosma@tcm.utcluj.ro; 3 Department of Anatomy, Universitat de Barcelona, 08036 Barcelona, Spain; xmoliva@icloud.com

**Keywords:** Achilles tendon revision surgery, cadaveric study, revision constructs, biomechanical testing, suture technique

## Abstract

Background: Achilles tendon re-rupture following operative repair remains a challenging complication, and biomechanical evidence guiding revision strategies is limited. The mechanical behavior of commonly used revision constructs has not been well characterized. The objective of this exploratory study was to provide a descriptive biomechanical characterization of commonly used Achilles tendon revision constructs, focusing on viscoelastic behavior, load-to-failure properties, and failure mechanisms under standardized loading conditions. Although limited by the absence of construct replication, this study provides hypothesis-generating biomechanical insight into the failure mechanisms of revision constructs, which may inform future comparative studies and surgical strategy selection. Methods: Four fresh-frozen human cadaveric lower limbs underwent standardized Achilles tendon transection with segmental excision to simulate revision conditions. Five revision techniques were evaluated: tensioned cross-lock Bunnell, Krakow, posterior tibial tendon (PTT) augmentation with Bunnell repair, double Kessler with circumferential running suture, and V–Y advancement combined with three simple sutures and double Kessler. All repairs were performed using No. 2 high-strength suturing by a single surgeon. Constructs underwent stress relaxation testing under a constant 100 N load followed by uniaxial load-to-failure testing. Mechanical parameters and failure modes were recorded. Results: All constructs demonstrated time-dependent stress relaxation. The tensioned cross-lock Bunnell repair retained the highest residual force during sustained loading. The PTT-augmented construct exhibited the highest load to failure among the constructs tested and failed at the tendon substance, whereas non-augmented repairs failed predominantly at the suture–tendon interface. The V–Y advancement construct failed at relatively low applied loads under the applied testing protocol. Conclusions: Achilles tendon revision constructs demonstrate distinct biomechanical behaviors. Augmented constructs exhibited higher resistance to tensile loading in this experimental setting and shifted failure away from the repair site, while non-augmented repairs were limited by suture–tendon interface strength. Given that each construct was tested only once and that one specimen was used sequentially for two repairs, the findings should be interpreted strictly as descriptive and hypothesis-generating, without any basis for comparative or inferential conclusions.

## 1. Introduction

The reported incidence of Achilles tendon re-rupture following primary operative treatment ranges from 1.5% to 5%, with male sex, younger age, pre-existing tendinopathy, and early postoperative mobilization identified as major risk factors [1,2,3]. Despite advances in surgical techniques, re-rupture remains a clinically significant complication with substantial functional consequences.

The Achilles tendon, formed by the confluence of the gastrocnemius and soleus tendons, serves as the primary plantarflexor of the ankle during gait. During late stance, when the ankle is dorsiflexed and the knee approaches full extension, tensile loads transmitted through the tendon may reach up to twelve times body weight. These extreme biomechanical demands, combined with the tendon’s limited intrinsic healing capacity, contribute to its vulnerability to failure [4].

The Achilles tendon dynamically adapts its force, stiffness, and functional behavior to the specific type, magnitude, and repetition of mechanical loading during an activity; however, when mechanical loading exceeds or is incongruent with its adaptive capacity, this results in compensatory biomechanical alterations, progression to tendinopathy, and eventual structural failure culminating in rupture [5,6,7,8].

Most Achilles tendon ruptures occur within a hypovascular zone located 2–6 cm proximal to the calcaneal insertion, where reduced perfusion compromises tissue regeneration and postoperative healing. This region remains particularly susceptible during revision surgery, where tissue quality is often further impaired by prior surgical trauma or degeneration [9,10].

In primary Achilles tendon rupture, surgical repair strategies typically aim to restore tendon continuity while preserving vascularity and minimizing soft tissue disruption. Commonly used techniques include open end-to-end repairs such as the Krakow or Bunnell configurations, as well as minimally invasive and percutaneous approaches, which have gained popularity due to reduced wound complications while maintaining comparable re-rupture rates [11,12,13,14]. Current consensus suggests that no single technique has demonstrated clear superiority, and choice of repair is often guided by surgeon preference and patient-specific factors, including activity level and tendon quality.

In contrast, revision Achilles tendon repair presents distinct biomechanical and clinical challenges. Re-rupture is frequently associated with compromised tendon integrity, scar formation, elongation, and segmental defects, which impair the ability of standard end-to-end repairs to restore native load transmission. As a result, revision strategies often require augmentation procedures, tendon transfers, or lengthening techniques to bridge defects and improve construct strength. These approaches alter both the geometry and mechanical behavior of the tendon, particularly with respect to stiffness, load distribution, and failure mechanisms.

It should also be noted that, although the present study aimed to simulate revision conditions through segmental tendon excision, the cadaveric specimens had no prior surgical intervention. Consequently, the model does not fully replicate the biological environment of true revision cases, where altered vascularity, adhesions, and degenerative changes may further influence mechanical behavior.

Indications for revision surgery include tendon re-rupture, persistent gapping—defined as a clinically or radiologically detectable separation between tendon ends (typically >5–10 mm) following primary repair due to elongation or mechanical insufficiency—and failure of wound or soft tissue healing [15]. While suture configuration plays a critical role in construct stability, the material properties of the suture itself have emerged as an equally important determinant of biomechanical performance. High-strength suture tapes have demonstrated superior resistance to cyclic loading and higher loads to failure compared with conventional sutures [16,17,18,19].

Currently, no standardized protocol exists for the surgical management of Achilles tendon re-rupture. Most revision strategies are extrapolated from techniques used in chronic ruptures and are tailored according to defect size and residual tissue quality. Larger defects frequently require augmentation through tendon transfers, lengthening procedures, or allograft reconstruction [15,20].

The purpose of this study was to evaluate the biomechanical resistance of commonly used suture techniques in Achilles tendon revision using a cadaveric model. We hypothesized that augmented and circumferentially reinforced revision constructs would exhibit distinct mechanical behaviors under tensile loading compared with non-augmented end-to-end repairs. Despite the wide range of available revision techniques, biomechanical data comparing their mechanical behavior under standardized loading conditions remain scarce, particularly in revision-specific settings. A substantial body of biomechanical research has investigated primary Achilles tendon repair techniques, including comparisons of suture configurations, materials, and fixation strategies under both cyclic and load-to-failure conditions. These studies have demonstrated that suture technique, pretensioning, and construct geometry significantly influence stiffness, gap formation, and ultimate failure strength. In particular, locking configurations such as the Krakow and Bunnell techniques have been shown to provide improved load distribution compared with non-locking repairs, while high-strength suture materials and tape constructs may further enhance mechanical performance under cyclic loading. However, most of these investigations have focused on acute midsubstance ruptures in controlled experimental settings and have relied on replicated constructs with statistical comparison. In contrast, biomechanical data addressing revision-specific conditions—characterized by segmental defects, altered tissue quality, and the need for augmentation or lengthening procedures—remain limited. The objective of the present study was therefore to provide a descriptive, exploratory biomechanical characterization of commonly used Achilles tendon revision constructs.

While prior biomechanical studies have predominantly focused on primary Achilles tendon repair using standardized and replicated constructs, the scientific novelty of this study lies in its focus on revision scenarios, the inclusion of heterogeneous construct types tested under identical conditions, and the evaluation of both viscoelastic behavior and failure modes in a revision-oriented model. This study was not designed to establish comparative superiority, but to generate mechanistic insights and hypotheses to guide future biomechanical and clinical investigations.

## 2. Materials and Methods

### 2.1. Specimen Preparation

Four fresh-frozen human lower limbs (two left, two right) were obtained through the body donation program of the University of Barcelona (Facultat de Medicina, Campus Hospital Clínic). All specimens were harvested from separate donors and tested within four weeks post mortem to preserve tendon mechanical properties. Prior to testing, specimens were thawed at room temperature for approximately 12 h to allow for complete soft tissue equilibration before preparation and mechanical testing. Donors had no known history of lower-limb surgery, trauma, systemic inflammatory disease, or Achilles tendon pathology. Macroscopic inspection confirmed intact tendons without calcifications or degenerative changes. The study was conducted in accordance with institutional ethical standards for human tissue research. All specimens were kept wrapped in saline-moistened gauze during preparation to minimize dehydration prior to mechanical testing.

### 2.2. Surgical Simulation

Four cadaveric lower limbs were available for testing. To allow for an evaluation of five distinct revision constructs within this exploratory design, one specimen was used sequentially for two different repair techniques, that is tensioned cross Bunnel followed by Krakow. The second repair was performed on a separate tendon segment after completion of the initial test. Each construct was therefore tested only once, and one specimen was used sequentially for two different repairs. This design precludes any form of replication and introduces potential specimen-dependent effects. Accordingly, no direct comparisons between constructs were intended, and the study was designed strictly as a descriptive, hypothesis-generating analysis.

**Figure 1:**
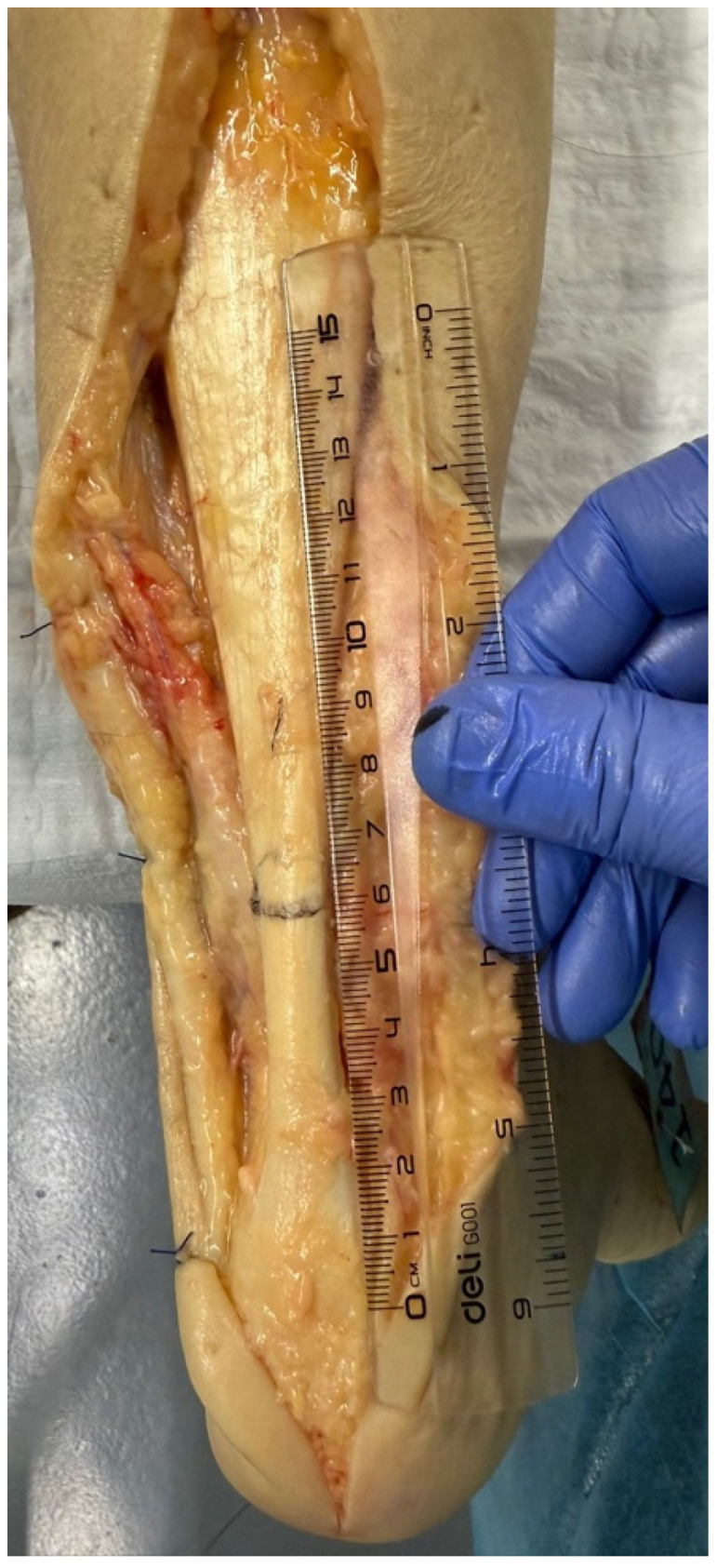
A representation of the incision mark at the 6 cm level from the calcaneal insertion.

A standardized medial paratendinous approach was performed in all specimens. After exposure, the Achilles tendon was transected transversely 6 cm proximal to the calcaneal insertion (Figure 1). To simulate revision conditions and compromised tissue quality, an additional 1 cm segment was excised proximally and distally, creating a reproducible defect.

**Figure 2:**
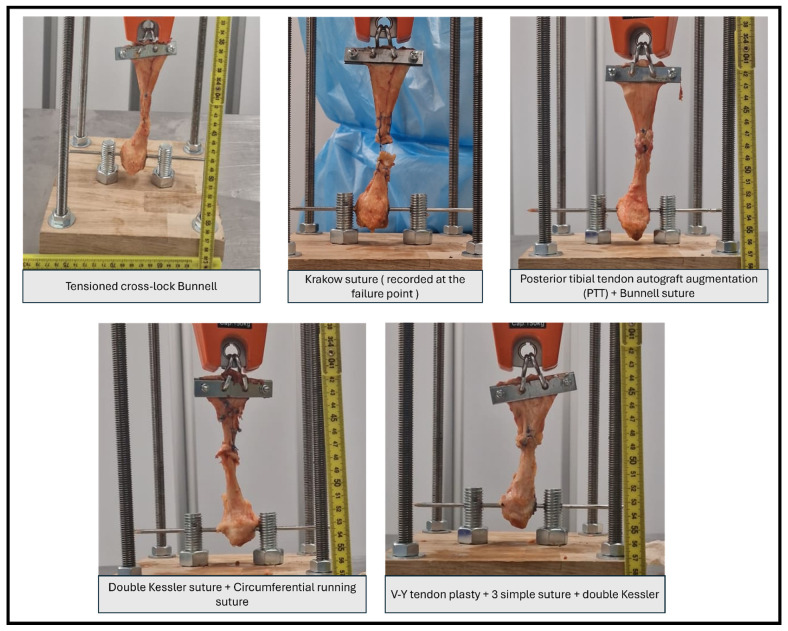
A representation of each sutured tendon anchored on the testing device.

**Figure 3:**
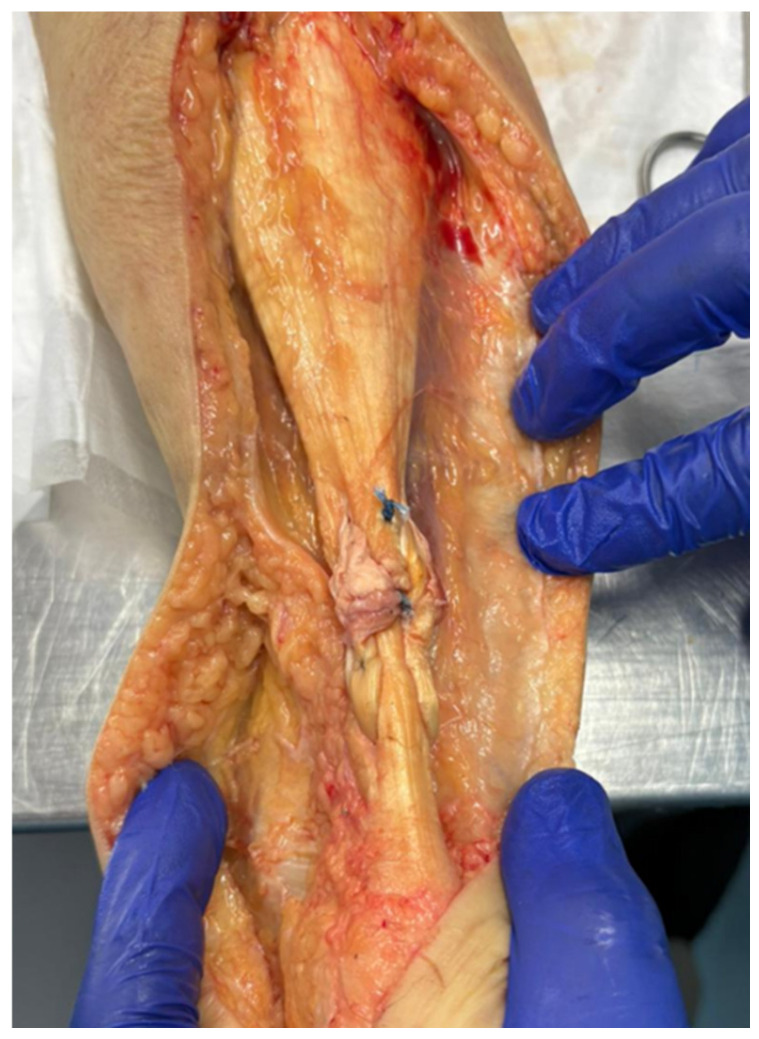
The PTT + Bunnell suture complex in vivo.

**Figure 4:**
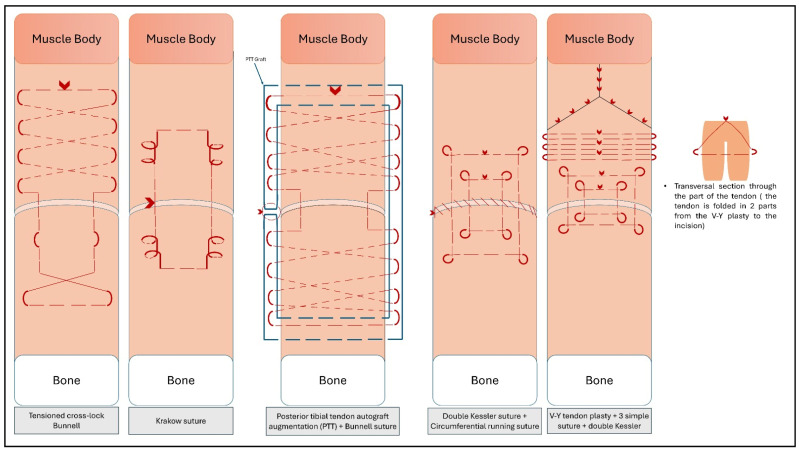
The pattern of each suture studied in order from left to right. The figure highlights a key technical feature of the V–Y tendon plasty construct: distal to the V–Y advancement, the tendon is folded longitudinally onto itself, creating a doubled tendon segment that is stabilized with three simple sutures before application of the double Kessler repair.

Five revision strategies were evaluated in an exploratory manner (Figure 2, Figure 3 and Figure 4):

1. Tensioned cross-lock Bunnell.
2. Krakow suture.
3. Posterior tibial tendon autograft augmentation combined with Bunnell repair.
4. Double Kessler with circumferential running suture.
5. V–Y advancement combined with three simple sutures and double Kessler.

All repairs were performed using No. 2 FiberWire by the same surgeon to reduce technical variability. Accordingly, the effects of alternative suture materials or different pretensioning protocols were not evaluated in the present study. The selected revision strategies were chosen to represent a spectrum of commonly employed biomechanical principles in revision Achilles tendon surgery, including end-to-end repair, circumferential reinforcement, tendon augmentation, and myotendinous advancement. Technique selection was based on reproducibility within a cadaveric model and local surgical practice rather than prevalence alone. More complex transfers, such as flexor hallucis longus reconstruction, were not included due to their additional osseous fixation requirements and limited standardization in cadaveric tensile testing.

Each construct was tested in a single specimen without replication, and one specimen underwent sequential testing of two repair techniques. This approach was intentionally adopted to allow for the inclusion of multiple constructs within a limited cadaveric sample; however, it represents a fundamental methodological limitation. Accordingly, the results are strictly descriptive and intended only for hypothesis generation, without statistical comparison or inference.

No histological or microstructural analysis was performed, as the study was designed to evaluate time-zero mechanical properties under controlled loading conditions rather than biological tissue response.

A cyclic loading protocol was not included in the present study; therefore, the mechanical evaluation was limited to time-zero stress relaxation behavior under constant load and subsequent load-to-failure testing. Consequently, parameters relevant to early postoperative conditions, such as gap formation, cumulative elongation, and fatigue resistance under repetitive loading, were not assessed.

### 2.3. Biomechanical Testing

A custom mechanical testing apparatus was used to apply uniaxial tensile loading. The device was constructed from four vertically oriented threaded metallic support rods reinforced with rigid oak stabilization plates to minimize deformation during loading. The system consisted of a rigid fixation frame with calcaneal anchoring and a dynamometer-based force measurement unit connected to the proximal tendon clamp. Tensile loading was generated using a custom linear actuator system driven by a NEMA 17 bipolar stepper motor (Stepperonline, New York, NY, USA) (holding torque approximately 0.36 Nm, 200 steps/revolution) coupled to a lead screw displacement mechanism, allowing controlled displacement-rate tensile testing. The motor-driven actuator mechanism was positioned centrally on the superior plate, allowing gradual and controlled application of tensile force on the dynamometer, respectively on the tendon. The motor–lead screw system was theoretically capable of generating tensile forces exceeding 500 N, which was above the maximum loads applied in the present study. The 100 N constant load and 240-s duration were selected to represent submaximal early postoperative tensile forces and to allow for the assessment of short-term viscoelastic behavior, consistent with prior cadaveric tendon studies. The calcaneus was rigidly fixed using a 4 mm Kirschner wire inserted transversely and secured to the testing frame to prevent rotation or displacement. The proximal tendon was clamped using a custom serrated grip connected to the dynamometer, ensuring uniform load distribution and minimizing slippage during testing. Calibration was performed using standardized weights prior to testing. Repeated calibration cycles performed before testing demonstrated stable and reproducible force measurements throughout the operational loading range. A preload of 10 N was applied, followed by preconditioning with five load–unload cycles at 50 N. Force measurements were obtained using a calibrated dynamometer with appropriate sensitivity for low-load tendon testing, and calibration stability was verified prior to each testing session. Load-to-failure testing was performed at a constant displacement rate of 20 mm/min, within the operational capabilities of the device. All specimens underwent a single freeze–thaw cycle prior to testing.

Cross-sectional area (CSA) measurements were obtained at the level of the repair site for each construct following completion of the suturing technique. Tendon width and thickness were measured using a digital caliper, and CSA was calculated assuming an elliptical geometry using the formula π × (width/2) × (thickness/2). All measurements were performed in a standardized fashion by the same investigator to minimize interobserver variability. Given the non-uniform morphology of the Achilles tendon, this method provides an approximation of the local cross-sectional area at the repair site and may not fully capture regional variations in tendon geometry.

Following stress relaxation testing, uniaxial tensile loading was applied until construct failure. Failure was defined as either complete rupture of the tendon or suture, or a sudden drop (>10%) in recorded force. During stress relaxation testing, displacement was held constant once the target load of 100 N was reached, and the decline in force over time was recorded. The force was recorded for 240 s, during which time it gradually declined due to internal structural rearrangements and viscoelastic relaxation. All mechanical trials were performed at room temperature (20 °C) with a relative humidity of 50%. Throughout specimen preparation and mechanical testing, tendon surfaces were periodically moistened with isotonic saline solution to reduce dehydration and preserve tissue viscoelastic properties; however, continuous hydration control (e.g., immersion or environmental chamber) was not employed. Load-to-failure testing was performed at a constant displacement rate of 20 mm/min, consistent with previously reported cadaveric tendon biomechanical studies. Mechanical parameters, including ultimate tensile strength, strain, and Young’s modulus, were calculated based on cross-sectional area measurements.

Young’s modulus was estimated from the linear region of the load–displacement curve, normalized to cross-sectional area and initial length. Given the exploratory design and specimen heterogeneity, these values are reported descriptively and do not represent intrinsic material properties.

Given the exploratory nature and limited sample size, data were analyzed descriptively.

## 3. Results

### 3.1. Specimen Geometry

Four fresh-frozen female cadaveric lower limbs were analyzed. The geometric characteristics of each repaired construct are summarized in Table 1. Considerable variability in tendon dimensions was observed across specimens. The largest cross-sectional area was measured in the double Kessler plus circumferential running suture (Surjet) construct (195.0 mm^2^), followed by the posterior tibial tendon augmentation with Bunnell repair (135.6 mm^2^).

### 3.2. Viscoelastic Behavior

**Figure 5:**
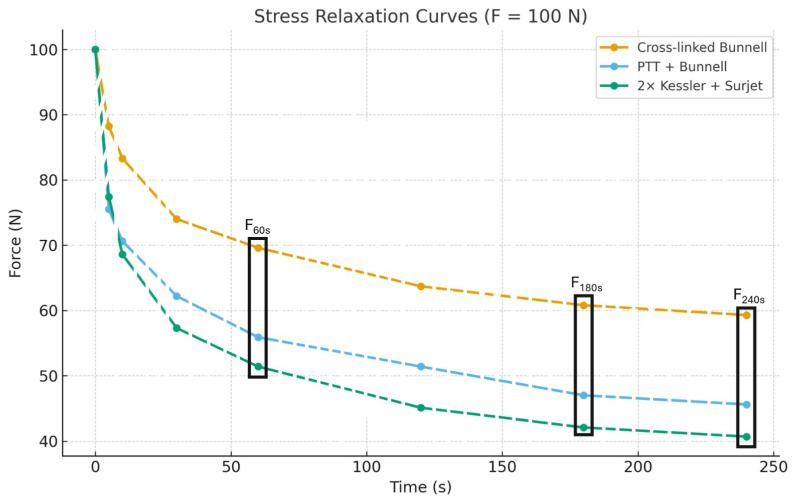
The stress relaxation curves represent the evolution over time of the retained force applied on the sutured elastic tendon after a constant initial force of 100 N, revealing the viscoelastic nature of the tissue.

Stress relaxation testing under a constant initial 100 N load was successfully completed for three constructs (Table 2, Figure 5). All tested repairs demonstrated a rapid decrease in retained force within the first 60 s, followed by a more gradual decline over time. The tensioned cross-lock Bunnell construct maintained the greatest residual force at 180 s among the constructs tested (60.8 N), whereas lower residual forces were observed in the augmented and circumferentially reinforced repairs.

### 3.3. Load-to-Failure Testing

Load-to-failure characteristics are summarized in Table 3. In this experimental series, load-to-failure values ranged from 47.5 N to 235.7 N across constructs. Augmented repairs exceeded 200 N, whereas certain non-augmented and advancement-based constructs failed below 100 N, reflecting substantial variability in initial mechanical resistance.

The highest load to failure observed in this series occurred in the posterior tibial tendon augmentation combined with Bunnell repair (235.7 N), followed by the double Kessler plus circumferential running suture (Surjet) construct (140.6 N). The Krakow and V–Y plasty-based repairs failed at lower applied loads in this experimental setup. The greatest elongation prior to failure was observed in the augmented construct (39 mm).

### 3.4. Failure Mode

**Figure 6:**
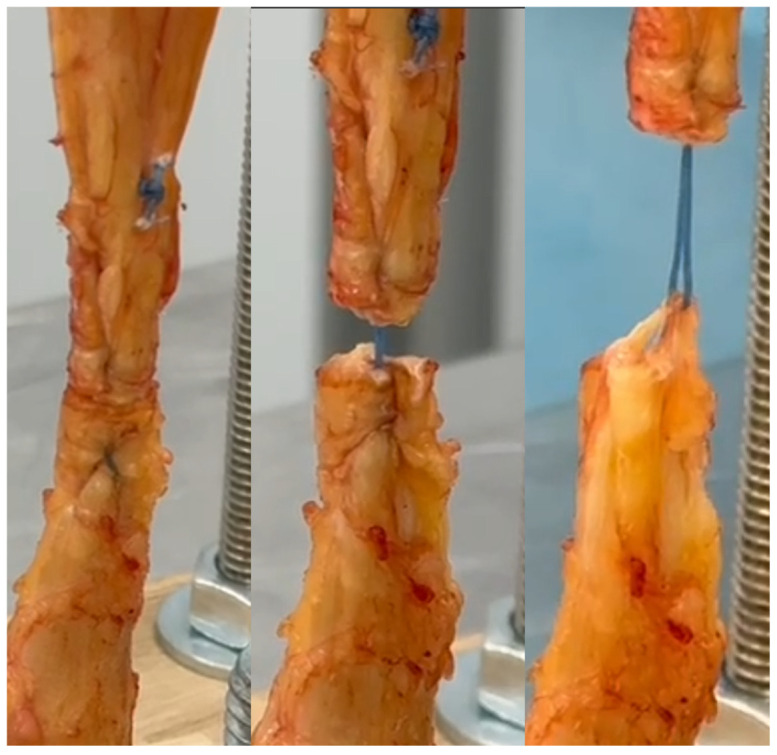
The evolution of the tensioned cross-lock Bunnell suture during the load-to-failure test. The thread is displaced through the tendon fibers, cutting through the tissue.

**Figure 7:**
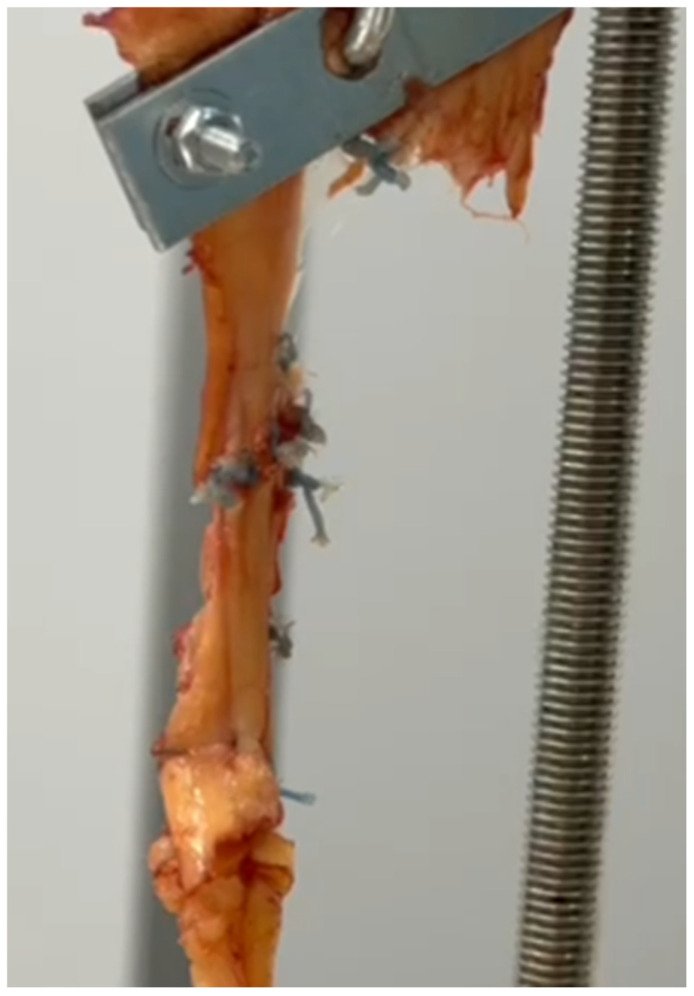
V–Y plasty rupture during the beginning of the viscoelastic test (the force applied is under 100 N).

Failure occurred at the suture–tendon interface in the tensioned cross-lock Bunnell (Figure 6) and Krakow repairs, whereas tendon rupture was observed in the augmented construct. The V–Y plasty-based repair failed at the myotendinous advancement during viscoelastic testing at an applied load of 47.5 N (Figure 7).

No statistical comparisons were performed due to the exploratory design. Given the absence of construct replication, all findings are reported descriptively without inferential statistical analysis.

Due to the absence of construct replication and variability in specimen geometry, full stress–strain curves are not presented, and mechanical parameters are reported as single-measure descriptors for each construct. Due to the absence of construct replication and the use of one specimen for sequential testing of two repairs, all results represent single-measure observations that may be influenced by specimen-specific factors. Accordingly, the findings are reported strictly as descriptive data without any inferential or comparative interpretation.

## 4. Discussion

To our knowledge, this is one of the few studies to focus specifically on the biomechanical behavior and failure modes of revision Achilles tendon constructs rather than primary repairs.

In the present study, tensile loading was applied using a custom-built uniaxial mechanical testing apparatus, consisting of rigid calcaneal fixation and a dynamometer-based loading system attached to the proximal tendon. The device is similar to those used in previous studies such as Swanson et al. and Macaluson et al. This configuration allowed for controlled application and continuous recording of tensile force along the anatomical axis of the Achilles tendon [21,22].

We acknowledge that commercially available universal testing systems provide highly standardized loading conditions and excellent intertrial repeatability. However, the custom-built apparatus used in the present study was specifically designed for cadaveric Achilles tendon testing in order to accommodate anatomical fixation constraints and minimize specimen slippage during tensile loading. The system incorporated rigid metallic fixation components, displacement-controlled actuator-driven loading, calibrated dynamometer-based force acquisition, and repeated calibration cycles performed prior to testing sessions to ensure stable and reproducible force measurements within the loading range investigated.

Similar custom or adapted loading systems have been reported in previous cadaveric tendon biomechanical studies where specimen geometry and fixation requirements limit the use of standard commercial testing frames [21,22]. Although the present device was not formally validated against a commercial universal testing machine, repeated calibration procedures and controlled loading conditions provided acceptable measurement consistency for exploratory cadaveric biomechanical evaluation. Nevertheless, the absence of formal inter-device validation remains a limitation of the present study and should be addressed in future investigations. Given the absence of replication, differences between constructs must be interpreted as specimen-specific observations rather than technique-dependent effects. Accordingly, comparisons between constructs should be interpreted descriptively and not as evidence of relative biomechanical superiority.

The present findings should be interpreted in the context of current clinical practice for revision techniques for Achilles tendon rupture, which remains largely individualized. In routine practice, revision strategy is typically selected according to tendon gap size, residual tissue quality, prior surgery, and surgeon preference, with augmentation, tendon transfer, or advancement procedures used when direct end-to-end repair is not feasible. Our results do not establish the superiority of any technique, but provide time-zero biomechanical observations that may help explain why augmented constructs may better tolerate tensile loading, whereas non-augmented repairs may remain limited by the suture–tendon interface.

Marked differences in construct geometry were observed, particularly in cross-sectional area. Augmented constructs, including posterior tibial tendon (PTT) augmentation and circumferentially reinforced repairs, demonstrated substantially larger cross-sectional areas compared with non-augmented end-to-end sutures. This increase in bulk is consistent with prior reports describing augmentation-related increases in tendon dimensions and may influence both initial stiffness and load distribution across the repair site. Previous work has reported a mean Achilles tendon cross-sectional area of approximately 49 mm^2^ in healthy tendons, highlighting the substantial geometric alteration introduced by augmentation relative to reported values for healthy Achilles tendons [23].

Although smaller cross-sectional areas in the present series were associated with suture–tendon interface failure in some non-augmented repairs, the exploratory design does not permit identification of a minimum geometric threshold for safe fixation. Cross-sectional area, tissue quality, augmentation, and suture configuration are interdependent in this model and cannot be analyzed separately.

This study does not support the definition of a minimum cross-sectional area or tissue quality index predictive of suture–tendon interface failure. No histological or quantitative tissue quality assessment was performed, and the lack of construct replication precludes threshold analysis.

All constructs demonstrated a characteristic stress relaxation pattern, reflecting the viscoelastic behavior of tendon tissue. Stress relaxation in tendon repair constructs reflects internal redistribution of load within the collagen–suture complex over time, resulting in a gradual decline in retained force despite constant elongation. In sutured constructs, the stress relaxation is influenced by suture configuration, tissue quality, and the mechanical interaction between the thread and the tendon tissue under tensile loading conditions. Differences between constructs suggest variation in compliance, with higher residual force indicating reduced early elongation under sustained load. The tensioned cross-lock Bunnell repair maintained higher residual force over time, whereas augmented and circumferentially reinforced constructs exhibited greater relaxation, indicating increased compliance. From a biomechanical perspective, increased compliance may facilitate load sharing and energy dissipation; however, it may also predispose to early gap formation at the repair site under sustained postoperative loading conditions. Although limited to a constant-load protocol, these findings indicate that viscoelastic properties may influence early construct stability in the postoperative period. These findings suggest that viscoelastic behavior may represent an additional determinant of construct performance beyond load-to-failure characteristics alone.

Although the present cadaveric model does not allow for direct extrapolation to clinical outcomes, these findings highlight the potential importance of viscoelastic properties in the early postoperative period, where time-dependent elongation may influence construct stability and tendon healing.

Load-to-failure testing revealed the highest ultimate load in the PTT-augmented Bunnell construct, which failed at the tendon substance rather than the suture–tendon interface. In contrast, both the Krakow and tensioned cross-lock Bunnell repairs failed at the level of the suture. These findings are broadly consistent with prior biomechanical studies showing that construct configuration and pretension influence repair stiffness and failure mode, and that stronger or augmented constructs may shift the weakest point away from the repair site. Compared with previously published studies reporting higher failure loads for the tensioned cross-lock Bunnell technique, the lower failure force observed in the present study may be explained by the revision-like defect model, compromised local tissue conditions, and localized stress concentration at the transverse suture segment, which may have acted as a focal cutting element rather than a load-distributing structure [24]. These findings do not imply superiority over more commonly used transfers such as the flexor hallucis longus, but rather support further biomechanical investigation of alternative graft sources.

Although the present study was not designed to define clinical thresholds, the observed range of failure loads provides a descriptive indication of construct behavior under tensile loading. Constructs failing below 100 N may be more susceptible to early mechanical compromise under tensile stress, whereas those exceeding 200 N demonstrated greater resistance in this time-zero setting. However, these values should not be interpreted as safe loading limits, as in vivo forces and biological healing responses are not replicated in this model.

Direct comparison with prior biomechanical research must be made cautiously. Most available cadaveric studies have evaluated primary midsubstance Achilles repairs using replicated constructs and averaged failure values, whereas the present study focused on revision-oriented techniques in a non-replicated exploratory model. Accordingly, our findings are more useful for identifying construct-specific mechanical patterns and failure mechanisms than for quantitative ranking against previously published techniques. This distinction is particularly relevant because revision surgery involves poorer tissue quality, segmental loss, and reconstructive strategies that are not fully represented in primary repair models.

The load-to-failure values observed in the present study are substantially lower than the forces sustained by the native Achilles tendon under physiological conditions, which have been reported to reach several thousand newtons during activities such as walking or running [25,26]. This discrepancy reflects the fundamental difference between intact tendon properties and the mechanical behavior of repair constructs at time zero. In the immediate postoperative setting, the strength of the repair is governed primarily by the suture–tendon interface and fixation technique, which represent the weakest link in the construct.

Previous cadaveric studies evaluating primary Achilles tendon repairs have reported load-to-failure values typically ranging between approximately 100 and 400 N, depending on suture configuration, material, and testing protocol. In this context, the values observed in the present study—particularly for non-augmented repairs—are consistent with the existing biomechanical literature. The higher load observed for the posterior tibial tendon-augmented construct may reflect improved load sharing and increased cross-sectional area, although this was accompanied by altered compliance characteristics.

Importantly, the present results should be interpreted as representing early postoperative construct strength prior to biological healing. In vivo, progressive tendon healing and remodeling contribute substantially to the restoration of mechanical integrity over time. In revision settings, where tissue quality is compromised and defects are present, initial construct strength may be further reduced, reinforcing the importance of protected loading during early rehabilitation.

Analysis of failure modes provides important insight into construct-specific vulnerabilities. Suture-level failure suggests inadequate stress distribution at the tendon–suture interface, whereas tendon-level rupture in augmented constructs indicates that fixation strength exceeded local tissue capacity. The early mechanical failure of the V–Y plasty-based construct under relatively low applied force suggests potential mechanical limitations under early tensile loading conditions of myotendinous advancement when used in isolation or combined with insufficient reinforcement. These findings emphasize the importance of matching revision strategy to defect size, tissue quality, and expected loading conditions [20,27].

The use of the posterior tibial tendon as an augmentation graft in Achilles tendon revision remains sparsely reported in the literature. Previous studies have primarily focused on flexor hallucis longus transfer, with limited exploration of alternative graft sources. The present findings suggest that PTT augmentation may represent a mechanically viable option in cases of substantial tendon deficiency, particularly when end-to-end repair alone is insufficient to restore continuity [20]. However, the biomechanical advantage of augmentation must be balanced against increased construct compliance and potential donor site considerations.

Fresh-frozen grafts have limitations compared to those of living tissue, including a lack of neuromuscular interaction and the absence of biological activity. Nevertheless, according to Moon et al. (2006) and Arnout et al. (2013), the refreezing of specimens has little or no effect on their biomechanical properties [28,29].

Interpretation of these findings should consider the exploratory nature of this study. Unlike prior biomechanical investigations that report averaged outcomes from multiple identical constructs, each repair technique in this study was represented by a single specimen. As such, comparisons with previously published data should be viewed as contextual rather than directly equivalent.

The principal limitation of this study is the evaluation of each revision construct in a single specimen, which precludes statistical comparison and limits generalizability. All specimens were derived from female donors, and although sex-related differences in tendon morphology and material properties have been reported, the limited sample size precluded meaningful subgroup analysis. Additionally, cadaveric testing does not replicate in vivo biological healing, neuromuscular loading, or postoperative rehabilitation forces. In addition, although specimens were intermittently moistened with saline during testing, strict hydration control was not maintained, and dehydration may have influenced the measured viscoelastic and mechanical properties. Cross-sectional area measurements and material property calculations were constrained by specimen heterogeneity and experimental resolution. Another limitation of this study is the absence of histological or tissue quality assessment. While such analyses could provide insight into tendon microstructure and degeneration, the use of fresh-frozen cadaveric specimens primarily allows for an evaluation of time-zero mechanical behavior rather than biological healing. Moreover, mechanical testing to failure compromises tissue integrity, limiting the feasibility and interpretability of subsequent histological analysis. Therefore, the present findings should be interpreted as purely biomechanical, without direct correlation to tissue remodeling or healing capacity. Despite these limitations, this study provides controlled descriptive insight into the mechanical behavior and failure mechanisms of revision constructs.

This study does not include full stress–strain curve analysis or elastic modulus profiling. Because each construct was tested in a single specimen with variable geometry, the calculated mechanical parameters should be interpreted as construct-specific descriptors rather than intrinsic material properties. Future studies with replicated samples would allow for more rigorous mechanical characterization, including curve-based analysis.

The observed failure modes may also depend on variables not directly tested in the present study, particularly suture material and pretension protocol. Prior biomechanical studies have shown that high-strength tape constructs may improve load distribution compared with round sutures, while pretensioning can modify construct stiffness and gap resistance. In this context, repairs that failed at the suture–tendon interface in the present series might exhibit a different failure pattern if broader tape-like sutures or alternative pretensioning strategies were used, potentially reducing localized cut-through. Conversely, increased stiffness or reduced elongation could also shift the weak point toward the tendon substance or augmentation segment. Because these variables were controlled rather than compared in the current experimental design, such effects remain hypothetical and warrant dedicated investigation.

The present viscoelastic findings should be interpreted within the limits of a single constant-load protocol. In a more rehabilitation-relevant cyclic loading model, constructs would likely exhibit additional time-dependent phenomena such as cumulative elongation, gap formation, progressive stiffness loss, and fatigue-related failure. Repairs limited by the suture–tendon interface may be particularly vulnerable to repeated loading, whereas augmented constructs may provide improved load sharing and delayed structural failure. At the same time, more compliant augmented constructs could also demonstrate greater cyclic creep. Because these responses were not directly tested in the current study, such implications remain speculative and should be examined in future cyclic loading experiments. An additional limitation is the absence of cyclic loading and direct assessment of gap formation to simulate early rehabilitation conditions. These parameters are clinically relevant, as repetitive submaximal loading may lead to progressive elongation, gap formation at the repair site, and eventual construct failure, particularly in the early postoperative period. Their absence limits the ability of the present model to predict construct performance under functional rehabilitation conditions. The stress relaxation test at 100 N provides only a simplified assessment of short-term viscoelastic response and does not reproduce repetitive postoperative loading. Future studies should incorporate cyclic loading protocols to assess gap formation, creep behavior, and fatigue resistance under clinically relevant rehabilitation conditions.

Beyond their mechanical role, augmentation techniques may also influence the biological environment of tendon healing. Although this cadaveric model does not permit direct assessment of cellular or histological processes, several potential mechanisms can be considered. Tendon graft augmentation may act as a scaffold facilitating cellular infiltration, neovascularization, and extracellular matrix remodeling. In addition, by increasing the cross-sectional area and redistributing load across the repair site, augmented constructs may reduce local strain concentrations, potentially promoting a more favorable biological healing response. Conversely, increased construct stiffness and bulk may alter physiological stress distribution and mechanotransduction, with uncertain effects on long-term tendon remodeling. The use of autologous grafts, such as the posterior tibial tendon, may further contribute to biological integration through viable tissue incorporation. However, these effects remain theoretical in the context of the present time-zero biomechanical study and should be investigated in future in vivo or histological studies.

The primary contribution of this study is not a comparative evaluation but a mechanistic characterization of revision constructs under controlled loading conditions. Given the limited biomechanical evidence available for revision Achilles tendon surgery, particularly regarding failure modes and viscoelastic behavior, this exploratory approach provides preliminary insights that may guide hypothesis generation and future study design.

Emerging strategies in tendon repair, including the use of advanced biomaterials and additive manufacturing techniques, may further enhance the mechanical and biological performance of revision constructs. Bioengineered scaffolds, synthetic augmentation materials, and three-dimensional printed structures have the potential to improve load distribution, provide a template for cellular infiltration, and allow for patient-specific construct design [30,31,32,33,34,35]. Such approaches may address some of the limitations of conventional suture-based repairs, particularly in cases of extensive tissue loss or poor tendon quality. However, these technologies remain largely experimental in the context of Achilles tendon revision, and standardized biomechanical and clinical data are currently limited. The present findings may therefore serve as a baseline for comparison in future studies evaluating biomaterial-augmented or additively manufactured repair strategies.

Future studies should evaluate larger replicated cohorts with standardized repair constructs and direct tissue quality assessment to determine whether geometric or structural thresholds can predict interface failure.

Future investigations should evaluate larger specimen cohorts and explore alternative graft options, including flexor hallucis longus, plantaris, peroneal tendons, and flexor digitorum longus. In cases of extensive tissue compromise, the biomechanical feasibility of dual-graft constructs warrants further study, as combined grafts may enhance load sharing and improve initial stability.

Future studies combining biomechanical testing with histological analysis or in vivo models may provide a more comprehensive understanding of how construct mechanics interact with tendon biology and healing processes.

## 5. Conclusions

This cadaveric study demonstrates distinct biomechanical behaviors and failure mechanisms among commonly used Achilles tendon revision constructs. In this series, failure loads ranged from approximately 50 N to over 230 N, with augmented constructs demonstrating the highest resistance to tensile loading. Posterior tibial tendon augmentation combined with a Bunnell suture demonstrated the highest load to failure within this limited experimental series and failed at the tendon level, whereas non-augmented repairs were more susceptible to suture–tendon interface failure. In relation to existing clinical practice, these findings support the view that augmentation may be mechanically advantageous in revision settings with compromised tissue quality, although no definitive treatment recommendations can be made from this exploratory model. Additionally, the three constructs evaluated under the stress-relaxation test presented viscoelastic behavior, with variations in relaxation profiles, reflecting the time-dependent mechanical behavior of repaired tendon tissue.

The construct incorporating V–Y lengthening showed limited mechanical durability under sustained load. These findings underscore the importance of tailoring revision strategies to tissue quality and defect characteristics. In addition to differences in load-to-failure characteristics and failure modes, all constructs demonstrated viscoelastic behavior, with variations in stress relaxation that may influence early mechanical stability.

While not generalizable, these findings highlight construct-specific mechanical vulnerabilities that may be relevant when selecting revision strategies in the context of compromised tendon quality.

The present findings are intended to characterize construct-specific mechanical behavior rather than to guide definitive clinical decision-making. These findings do not establish the superiority of any construct and should be interpreted strictly within the limitations of this exploratory cadaveric model; they are intended to inform future, adequately powered biomechanical and clinical studies. Given the absence of construct replication and the sequential use of one specimen, these findings should be interpreted strictly as descriptive and hypothesis-generating, and should not be used to support comparative or clinical conclusions.

## Figures and Tables

**Table 1:** Geometric characteristics of Achilles tendon revision constructs.

Parameter	Tensioned Cross-Lock Bunnell	Krakow	PTT Augmentation + Bunnell	Double Kessler + Circumferential Running Suture	V–Y Plasty + Simple Sutures + Double Kessler
Width (mm)	10.3	10.9	15.5	15.7	12.5
Thickness (mm)	7.6	7.3	11.2	15.8	11.8
Cross-sectional area (mm^2^)	62.0	62.1	135.6	195.0	116.0
Initial length (mm)	107	102	96	67	95

**Table 2:** Stress relaxation behavior under constant 100 N load.

Time Point	Tensioned Cross-Lock Bunnell	PTT Augmentation + Bunnell	Double Kessler + Circumferential Running Suture
Force at 0 s (N)	100.0	100.0	100.0
Force at 5 s (N)	88.2	75.5	77.4
Force at 10 s (N)	83.3	70.6	68.6
Force at 30 s (N)	74.0	62.2	57.3
Force at 60 s (N)	69.6	55.9	51.4
Force at 120 s (N)	63.7	51.4	45.1
Force at 180 s (N)	60.8	47.0	42.1
Force at 240 s (N)	59.3	45.6	40.7
Residual force at 180 s (N)	60.8	47.0	42.1
Relaxation rate 0–60 s (N/s)	0.50	0.74	0.81
Relaxation rate 0–180 s (N/s)	0.22	0.29	0.32
Relaxation rate 0–240 s (N/s)	0.17	0.23	0.25
Relaxation at 60 s (%)	30.4	44.1	48.6
Relaxation at 180 s (%)	39.2	53.0	57.9
Relaxation at 240 s (%)	40.7	54.4	59.3

**Table 3:** Load-to-failure characteristics of Achilles tendon revision constructs.

Parameter	Tensioned Cross-Lock Bunnell	Krakow	PTT Augmentation + Bunnell	Double Kessler + Circumferential Running Suture	V–Y Plasty + 3 Simple Sutures + Double Kessler
Load to failure (N)	132.8	66.6	235.7	140.6	47.5
Ultimate tensile strength (N/mm^2^)	2.14	1.07	1.73	0.72	0.40
Displacement at failure (mm)	122	110	135	88	101
Elongation at failure (mm)	15	8	39	21	6
Strain at failure (%)	14	7	40	31	6
Young’s modulus (N/mm^2^)	15.3	15.3	4.3	2.3	6.6
Failure mode	Suture	Suture	Tendon	Tendon	V–Y plasty

## Data Availability

Data available on request due to restrictions eg privacy or ethical The data presented in this study are available on request from the corresponding author. The data are not publicly available due to ethical and institutional restrictions related to the use of human cadaveric specimens.
